# 

**Published:** 1891-04-18

**Authors:** 


PRICE ONE PENNY.

No. 238, Vol. X.—April 18th, 1891.

[Registered at the General Post Office as a Newspaper for transmission in the United Kingdom and Abroad.]

WITH EXTRA NURSING SUPPLEMENT.
Per Annum, 6s. 6d.; post free.
Monthly Parts, 6d.; post free 8d.
Ditto per Annum, 8s., post free.

### THE HOSPITAL

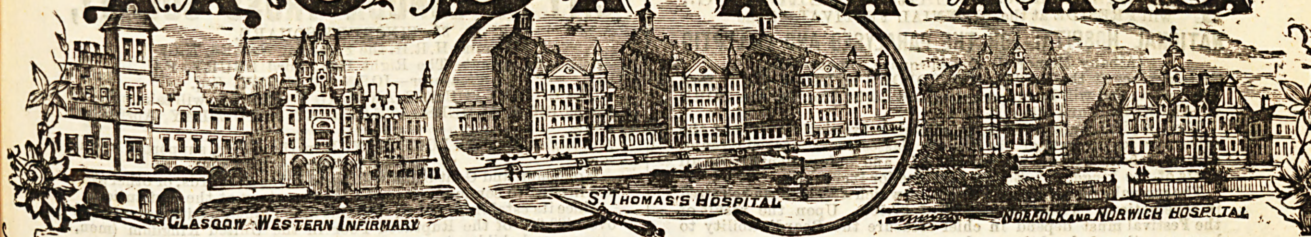

A WEEKLY JOURNAL OF
Science, Medicine, Nursing and Philanthropy.

### SATURDAY, APRIL 18th, 1891.

Tract Ninety-one: An Oxford Object Lesson - 25

Passing Topics.—A Good Example - 26

The Doctor's Armchair.—Professional Labels - 27

The Old Family Doctor - 28

Everybody's Page - 29

Notes and News - 30

Annotations.—Lord Hartington and the Derby Hospital—A "Specialist" Disturbance at Cheltenham—The Older Nurses - 32

The Radcliffe Infirmary, Oxford - 35

General Charities—Scraps and Gleanings - 31

The Practitioner's Mirror and Retrospect:—

MEDICINE.—Cold Baths in Typhoid Fever - 33

SURGERY.—Ingleby Lectures: Lecture II.—The Injuries of Bones - 34

THERAPEUTICS.—Bassorin Paste - 35

The Student of Medicine.—Lectures: Their Use and Abuse (*concluded*)—Students' Medical Societies—Appointments—Vacancies—Pass Lists.

### EXTRA SUPPLEMENT—THE NURSING MIRROR.

En Passant.—Roman Catholic Nurses—Kent Institution—Short Items—The Late Miss Fisher—Bristol Nurses' Society—Northern Nursing Association—New Inventions—An Extraordinary Report - xiii
Male Nurses.—By One of Them (*concluded*) - xiv
The Eastern Hospital Scandal - xv
For Reading to the Sick.—Happiness - xv
Death in Our Ranks - xv
Princess Christian's Daughter - xvii
The Nurses' Bookshelf.—Massage—A Health Resort—A Medical Lexicon—Nursing in Germany—The National Pension Fund - xvi
Everybody's Opinion.—The Second Thousand—The Eight Hours Bill, xvi.; Cruelty—A Nurse's Hours—Sanitas - xvii
Appointments—Notes and Queries - xvii
Off Duty.—Catherine Fidelis - xviii
To a Nurse - xviii
Amusements and Relaxation - xviii

